# Evaluation of Yanagihara facial nerve grading system based on a muscle fiber analysis of human facial muscles

**DOI:** 10.1007/s00405-019-05462-0

**Published:** 2019-05-10

**Authors:** Kumiko Sekikawa, Hiroshi Moriyama, Hidenobu Miyaso, Takuya Osada, Ryuichi Ueno, Naruhito Otsuka, Masahiro Itoh

**Affiliations:** 1 Department of Anatomy, Tokyo Medical University, Tokyo, Japan; 2 Department of Anatomy, Showa University School of Medicine, 5-8, Hatanodai 1, Shinagawa-ku, Tokyo, 142-8555 Japan; 3 Rehabilitation Center, Tokyo Medical University Hospital, Tokyo, Japan

**Keywords:** Facial muscle, Muscle fiber, Morphometry, Yanagihara facial nerve grading system

## Abstract

**Purpose:**

We morphometrically analyzed human facial muscles, and evaluated the Yanagihara facial nerve grading system using our data.

**Methods:**

We used 15 types of human facial muscle, 2 types of masticatory muscle and 2 types of skeletal muscle. The materials were obtained from 11 Japanese male cadavers aged 43–86 years. We counted the muscle fibers and measured the transverse area of the muscle fibers (TAMF), and then calculated the number of muscle fibers (NMF) per mm^2^ and the average TAMF.

**Results:**

We found a significant correlation between average TAMF and NMF (*r* = − 0.70; *p* < 0.01). We classified facial muscles into three types based on the correlational results. Type A had a low average TAMF and high NMF. Type C had a high average TAMF and low NMF. Masticatory and skeletal muscles were characterized as Type C. Type B was intermediate between Types A and C.

**Conclusions:**

Pathological changes in the facial muscles in facial nerve palsy seem to vary according to the type of facial muscle, because each facial muscle has a unique fiber-type composition. As the nine discrete facial expressive states evaluated in the Yanagihara system involve all three facial muscle types of our classification, the Yanagihara system is an outstanding system for grading facial nerve palsy in terms of the facial muscle morphology.

## Introduction

Several grading systems for the assessment of facial nerve function using gross or regional scales have been proposed. The Yanagihara facial nerve grading system, reported by Yanagihara [1], was developed in Japan as a representative regional scale, and was standardized in Japan and in some other countries for grading facial function. The Yanagihara system measures 10 separate aspects of different facial functions. Each function is scored as 0 (complete palsy), 2 (partial palsy), or 4 (nearly normal), with a maximum total score of 40. The total score provides information on the grade of facial nerve dysfunction.

The facial muscles differ from one another in morphological aspects. Researchers have reported that clinicians must consider the diversity of facial muscles when planning treatments for facial paralysis [2, 3]. In this study, we morphometrically analyzed human facial muscles, and evaluated the Yanagihara facial nerve grading system using our data.

## Materials and methods

The materials were obtained from 11 Japanese male cadavers aged 43–86 years (average 71.8). Sex is reflected in various biological characteristics of human organs [4]. With regard to the skeletal muscles, Miller et al. reported sex differences in muscle fiber characteristics [5]. Smith and Mittendorfer also reported that differences in body composition between the sexes are evident from infancy but become most marked after puberty and persist into old age [6]. Moreover, as muscles are innervated by nerves, it is possible that sex differences in neurodegeneration influence sexual dimorphism of the muscle morphology. We used only male cadavers for reasons of the sexual dimorphism in the skeletal muscle described above, and clarified the characteristics of facial muscles without considering the effect of sexual dimorphism. All the cadavers were donated with the individuals’ consent. The muscle materials were as follows: frontal belly of occipitofrontalis, orbital part of orbicularis oculi, palpebral part of orbicularis oculi, levator labii superioris alaeque nasi, levator labii superioris, zygomaticus minor, levator anguli oris, zygomaticus major, buccinator, risorius, orbicularis oris, depressor anguli oris, depressor labii inferioris, mentalis and platysma (facial muscles), temporal and masseter (masticatory muscles), biceps brachii and tibialis anterior (skeletal muscles). All materials were resected together with skin and connective tissue. We conducted this research in accordance with the regulations concerning autopsies and the preservation of corpses, and concerning their donation for medical and dental education. In no case was there a history of neuromuscular disease such as myopathy or facial palsy, or of treatment with toxic agents or irradiation therapy. Each cadaver had 20 teeth or more (mastication capacity standard), and they supported themselves in daily life (biceps brachii capacity standard). The causes of death did not directly or indirectly influence the muscular or nervous systems, so we considered the dissected muscles to be normal. The preparation of sections involved fixation, washing, dehydration, embedding, and sectioning, as described in our previous report [7]. All the cadavers were fixed with a 10% solution of formalin (3.7% formaldehyde) within 24 h postmortem. After resection, the muscle samples were immersed in a 10% solution of formalin (3.7% formaldehyde) for at least 1 week. The solution was changed once in the first 30–60 min, and again later if necessary. The formalin-fixed materials were then transferred, without washing, to the secondary fixative and stored at room temperature for 2 weeks. The solution was changed if it precipitated or became turbid. After this, the fixation was continued at 37 °C for an additional week. The volume of fixative used was at least ten times the volume of the specimens. During this process, the materials were fixed with pins at four corners of a board. After washing, dehydration, and celloidin embedding, we cut 15-μm sections and stained them with hematoxylin and eosin (H&E).

### Morphometry

**Fig. 1:**
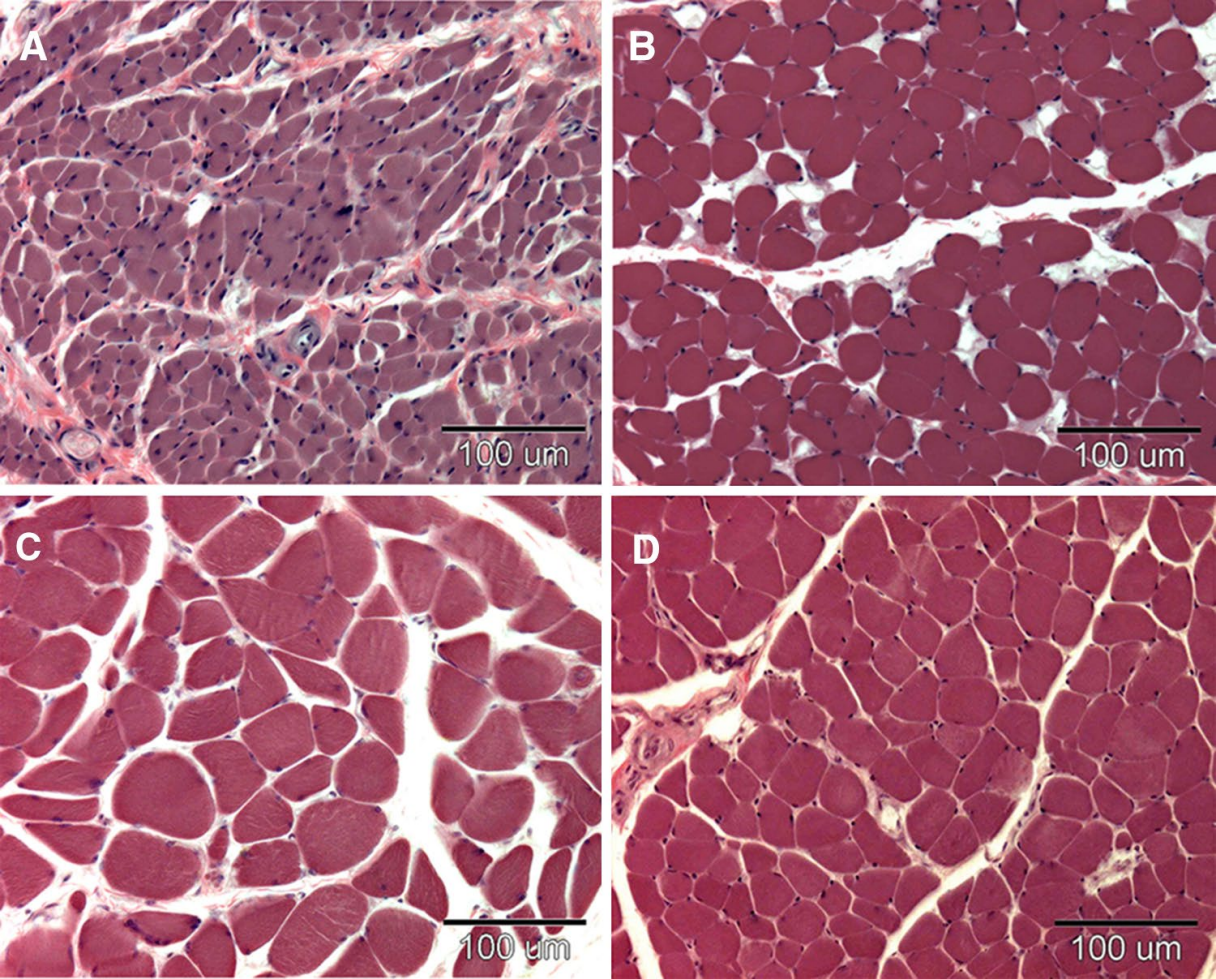
High-power views of the muscle fibers stained with H&E. 80-year-old man; scale bar = 100 μm. **a** Muscle fibers in the orbital part of the orbicularis oculi (Type A). **b** Muscle fibers in the buccinator (Type B). **c** Muscle fibers in the platysma (Type C). **d** Muscle fibers in the masseter (masticatory muscle)

We observed each microscopic section at low power, and covered the entire area of the distributed muscle fibers in the section by moving the eyepiece grid vertically and horizontally, as described in our previous report [8]. We confirmed that we could distinguish muscle fiber structures from other tissues with both a computer and the naked eye in every grid. We counted the muscle fibers and measured the transverse area of the muscle fibers in a square eyepiece grid at high power (Fig. 1), and then calculated the number of muscle fiber per mm^2^ (NMF) and the average transverse area of the muscle fibers (TAMF). To avoid duplicate counts, we counted and measured all muscle fibers on the side of the grid that did not contact with the other grids. In the case of grids adjacent to other grids, we counted and measured only the muscle fibers on the lower right side of the grid, not those on the upper left side. We used a microscope in transmitted light mode (BX50, Olympus, Tokyo, Japan) equipped with a high-resolution digital camera (ColorView12, Soft Imaging System, Münster, Germany), a motorized XYZ stage (Märzhäuser, Wetzlar-Steindorf, Germany), a stage controller (Märzhäuser, Wetzlar-Steindorf, Germany), and a computer (Precision 530, Dell, Austin, TX, USA) with analysis software (analySIS 3.0, Soft Imaging System, Münster, Germany) for storing data on-line, calculations, and statistical analyses.

### Statistical analyses

All statistical analyses were performed using JMP statistical software version Pro 13.0 (SAS Institute Inc. Cary, NC, USA) on an Apple Macintosh personal computer.

Researchers have studied the shrinkage of embedding materials, and found that celloidin and plastination embedding result in less shrinkage (around 10%) than paraffin and other materials [9]. Therefore, although we measured every muscle fiber, we calculated the transverse area of the muscle fibers after excluding data far from the median (15%) due to shrinkage.

The specimens used were sampled randomly. Two variables (*X* the average transverse area of the muscle fibers, and *Y* the number of muscle fibers per mm^2^) indicated a bivariate normal distribution. Thus, we calculated the coefficient of correlation (Pearson product moment; hereafter abbreviated as *r*) between the average transverse area of the muscle fibers and the number of muscle fibers per mm^2^.

## Results

We estimated the average transverse area of the muscle fibers (TAMF) and the number of muscle fiber per mm^2^ (NMF) in the facial muscles, masticatory muscles and skeletal muscles (Table 1). The average TAMF for the orbital part of orbicularis oculi muscles was 231 ± 95 (mean ± SD) μm^2^, and this value was the smallest among the 15 types of facial muscle. The average TAMF for platysma muscles was 610 ± 63 μm^2^, and this value was the largest. The average TAMFs for platysma, levator anguli oris and risorius muscles were approximately average among the masticatory and skeletal muscles, and were around three times as much as the average TAMF for the orbital part of orbicularis oculi muscles (Fig. 1a, c, d). Regarding the number of muscle fibers per mm^2^, NMF for the orbital part of orbicularis oculi muscles was 2210 ± 462, and this value was the largest of the 15 types of facial muscle. NMF for platysma muscles was the smallest, at 1061 ± 137. NMF for platysma muscles was half that for the orbital part of orbicularis oculi muscles (Fig. 1a, c).

**Table 1:** Comparison of the transverse area of muscle fibers and the number of muscle fibers in facial muscles, masticatory muscles, and skeletal muscles

Muscles	Area	Number
Facial muscles of Type A		
Frontal belly of occipitofrontalis	319 ± 130	1346 ± 386
Orbital part of orbicularis oculi	231 ± 95	2210 ± 462
Palpebral part of orbicularis oculi	282 ± 77	1410 ± 370
Levator labii superioris alaeque nasi	326 ± 107	2145 ± 369
Orbicularis oris	296 ± 68	1678 ± 280
Facial muscles of Type B		
Levator labii superioris	426 ± 91	1390 ± 269
Zygomaticus minor	441 ± 133	1237 ± 334
Zygomaticus major	472 ± 136	1258 ± 378
Buccinator	450 ± 109	1177 ± 258
Depressor anguli oris	467 ± 78	1253 ± 107
Depressor labii inferioris	431 ± 149	1330 ± 419
Mentalis	448 ± 116	1603 ± 364
Facial muscles of Type C		
Levator anguli oris	606 ± 194	1388 ± 330
Risorius	544 ± 141	1138 ± 346
Platysma	610 ± 63	1061 ± 137
Masticatory muscles		
Temporal	602 ± 230	885 ± 295
Masseter	697 ± 232	1209 ± 416
Skeletal muscles		
Biceps brachii	723 ± 302	1180 ± 460
Tibialis anterior	600 ± 250	1159 ± 468

Area indicates the average transverse area of muscle fibers (μm^2^)

Number indicates the number of muscle fibers per mm^2^

Each value is the mean ± SD (*n* = 11)

**Fig. 2:**
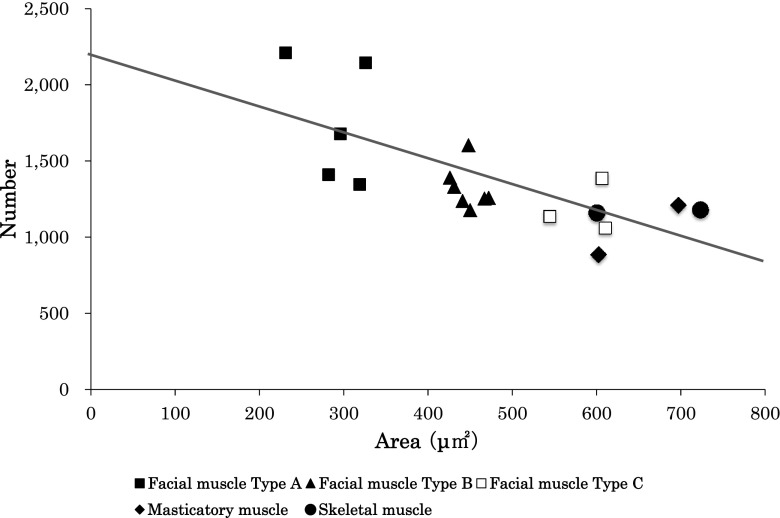
Relationship between the transverse area of muscle fibers and the number of muscle fibers. Area indicates the average transverse area of muscle fibers (μm^2^). Number indicates the number of muscle fibers per mm^2^. Each value is the mean (*n* = 11). Black squares indicate the values in the group of Type A facial muscles. Black triangles indicate the values in the group of Type B facial muscles. White squares indicate the values in the group of Type C facial muscles. Black diamonds indicate the values in the group of masticatory muscles. Black circles indicate the values in the group of skeletal muscles. Scatter diagram of the facial, masticatory and skeletal muscles shows a regression analysis of the average transverse area of the muscle fibers and the number of muscle fibers per mm^2^ (*r* = − 0.70; *p* < 0.01)

We calculated the coefficient of correlation (*r*) between the average TAMF and NMF, and found a significant correlation (*r* = − 0.70; *p* < 0.01) (Fig. 2). Next, we classified the facial muscles into three types, based on our correlational results (Table 1, Fig. 2). Type A had a low average TAMF and high NMF. Type C was characterized by a high average TAMF and low NMF. Masticatory and skeletal muscles were all characterized as Type C, among the facial muscles. Type B was intermediate between Types A and C. The frontal belly of occipitofrontalis, orbital part of orbicularis oculi, palpebral part of orbicularis oculi, levator labii superioris alaeque nasi and orbicularis oris were Type A, while Type B comprised the levator labii superioris, zygomaticus minor, zygomaticus major, buccinator, depressor anguli oris, depressor labii inferioris and mentalis. The levator anguli oris, risorius and platysma belonged to Type C.

## Discussion

The facial muscle system is distinct from the other skeletal muscles, in that it comprises flat, strap-like muscle sheets close to the skin with muscle fibers interdigitating or ending in the skin. The facial muscles have very short tendons, if any, are not enveloped by a fascia, and typical muscle bellies are not observed.

Researchers have studied the morphology of many kinds of skeletal muscle by calculating the muscle fiber number and area—the same method used in this study. However, there were few reports of muscle fiber numbers and areas for the facial muscle. It was reported that the muscle fiber number decreases in the case of muscle atrophy [10, 11]. The skeletal muscle volume in patients with sarcopenia was recently measured using CT, MRI and DXA (dual-energy X-ray absorptiometry). However, these noninvasive methods overestimated the skeletal muscle volume because they included the connective tissue, adipose tissue, extracellular fluid, and so on, in the intercellular spaces of muscle cells. The true skeletal muscle volume is calculated as the total number of muscle fibers × the average transverse area of muscle fibers. Thus, the number and the area of the muscle fibers are indispensable for estimating the facial muscles. We classified facial muscles by correlating of the number of muscle fibers with the average transverse area of muscle fibers. No similar study has been reported. Therefore, we consider that our morphometrical classification without using histochemical techniques is of value as a fresh viewpoint.

Happak et al. [2] and Freilinger et al. [3] reported that the facial muscles could be classified into three groups according to their relative proportions of fiber types: (1) phasic muscles with low percentages of slow type 1 fibers; (2) intermediate muscles with medium percentages of slow type 1 fibers; and (3) tonic muscles with high percentages of slow type 1 fibers. Our classification of the facial muscles agrees approximately with the above three groups, except for the frontal belly of the occipitofrontalis. The facial muscles of Types A, B, and C in our classification corresponded to (1) phasic muscles (2) intermediate muscles, and (3) tonic muscles, respectively.

Next, we examined the contraction of facial muscles to produce nine discrete facial expressive states, as evaluated in the Yanagihara system, excluding the “at rest” state (Table 2). The facial muscles used in expressive movements are as follows: (1) Type A for wrinkle forehead, close eyes normally, close eyes forcefully, close eyes on the involved side only, wrinkle nose, blow out cheeks and whistle; (2) Type B for blow out cheeks, grin and depress lower lip; and (3) Type C for grin. Thus, these nine discrete facial expressive states evaluated in the Yanagihara system involve all three types of facial muscles.

**Table 2:** Relationship between nine discrete facial expressive states evaluated in the Yanagihara system and the associated contracting facial muscles

Expressive movements	Muscles	Type
Wrinkle forehead	Frontal belly of occipitofrontalis	A
Close eyes normally	Palpebral part of orbicularis oculi	A
Close eyes forcefully	Palpebral part of orbicularis oculi	A
	Orbital part of orbicularis oculi	A
Close eyes on the involved side only	Palpebral part of orbicularis oculi	A
	Orbital part of orbicularis oculi	A
Wrinkle nose	Levator labii superioris alaeque nasi	A
	Procerus*	(A)
	Nasalis *	(A)
Blow out cheeks	Buccinator	B
	Orbicularis oris	A
Whistle	Orbicularis oris	A
Grin	Levator labii superioris	B
	Zygomaticus minor	B
	Zygomaticus major	B
	Depressor labii inferioris	B
	Levator anguli oris	C
	Risorius	C
Depress lower lip	Depressor anguli oris	B

*No specimens in this study. (A) is classified on the criteria of Freilinger et al. [3]

Skeletal muscle fiber types can have a profound impact on muscle diseases, including certain muscular dystrophies and sarcopenia, the aging-induced loss of muscle mass and strength. Talbot and Maves [12] reported that the components of different muscle fiber types mediate their susceptibility or resistance to disease. For example, muscle disorders with effects on specific skeletal muscle fiber types include the following: (1) muscle inactivity by spinal cord injury or bed rest appears to most strongly induce type 1 fiber atrophy, accompanied by a fiber-type shift from type 1 and 2A fibers to type 2X [13, 14]; (2) sarcopenia is characterized by a selective reduced size and greater atrophy of type 2 fibers [15, 16]; (3) in Duchenne muscular dystrophy, type 2 fibers are the first to degenerate and are eventually lost, whereas type 1 fibers are affected relatively late [17–19]. These reports suggest that some muscle diseases may be treated by shifting the fiber-type characteristics, depending on the disease. Thus, the muscles influenced by pathological changes and aging depend upon the fiber-type composition (type 1, 2A, or 2X). Regarding the facial muscles, Jergović et al. reported that the fiber-type composition changed after facial nerve injury and repair in an animal experimental study [20]. Moreover, Cheng et al. reported that elderly human subjects showed a decreased cross-sectional area of type IIA fibers compared to young subjects; the relative content of type IIA fibers decreased with increasing age, while the relative contents of type I and IIX fibers did not change [21]. Although there are few reports on the specific fiber types affected by facial nerve palsy, we assume that the pathological changes to facial muscles (muscle atrophy, muscle weakness, etc.) in facial nerve palsy depend upon the facial muscle type as classified in this study. As our results above show, the facial muscle types in this study (Types A, B and C) have unique compositions of fiber-types 1, 2A and 2X. We propose that all three facial muscle types in our classification should be examined in facial nerve palsy. As the nine discrete facial expressive states evaluated in the Yanagihara facial nerve grading system involve all three facial muscle types (Table 2), and the Yanagihara system is an outstanding grading system for facial nerve palsy in terms of the facial muscle morphology.

## Conclusion

Pathological changes in the facial muscles in facial nerve palsy seem to vary according to the type of facial muscle, as these muscles have distinct fiber-type compositions. The facial muscles of Types A, B, and C in our classification corresponded to (1) phasic muscles with low percentages of slow type 1 fibers (2) intermediate muscles with medium percentages of slow type 1 fibers, and (3) tonic muscles with high percentages of slow type 1 fibers. The nine discrete facial expressive states evaluated in the Yanagihara facial nerve grading system involve all three facial muscle types in our classification, making it an outstanding grading system for facial nerve palsy in terms of the facial muscle morphology.
